# Role of pH in Regulating Cancer Pyrimidine Synthesis

**DOI:** 10.3390/jox12030014

**Published:** 2022-07-06

**Authors:** Saad Saeed Alqahtani, Tomas Koltai, Muntaser E. Ibrahim, Adil H. H. Bashir, Sari T. S. Alhoufie, Samrein B. M. Ahmed, Daria Di Molfetta, Tiago M. A. Carvalho, Rosa Angela Cardone, Stephan Joel Reshkin, Abdelhameed Hifny, Mohamed E. Ahmed, Khalid Omer Alfarouk

**Affiliations:** 1 Department of Pharmacy Practice, College of Pharmacy, Jazan University, Jazan 45142, Saudi Arabia; ssalqahtani@jazanu.edu.sa; 2 Pharmacy Practice Research Unit, College of Pharmacy, Jazan University, Jazan 45142, Saudi Arabia; 3 Via Pier Capponi 6, 50132 Florence, Italy; tkoltai@hotmail.com; 4 Department of Molecular Biology, Institute of Endemic Diseases, University of Khartoum, Khartoum 11111, Sudan; mibrahim@iend.org (M.E.I.); derma55@yahoo.com (A.H.H.B.); 5 Medical Laboratories Technology Department, College of Applied Medical Sciences, Taibah University, Medina 42353, Saudi Arabia; shoufie@taibahu.edu.sa; 6 Department of Biosciences and Chemistry, College of Health, Wellbeing and Life Sciences, Sheffield Hallam University, Sheffield S1 1WB, UK; samrein.ahmed@shu.ac.uk; 7 Department of Biosciences, Biotechnologies, and Biopharmaceutics, University of Bari, 70126 Bari, Italy; daria.dimolfetta@uniba.it (D.D.M.); tiagomac94@gmail.com (T.M.A.C.); rosaangela.cardone@uniba.it (R.A.C.); stephanjoel.reshkin@uniba.it (S.J.R.); 8 Faculty of Medicine, Al-Azhar University, Cairo 11651, Egypt; hameedhifnyinmadina@gmail.com; 9 Research Center, Zamzam University College, Khartoum 11123, Sudan; rector@zamzam.edu.sd; 10 Alfarouk Biomedical Research LLC, Temple Terrace, FL 33617, USA; 11 Hala Alfarouk Cancer Center, Khartoum 11123, Sudan

**Keywords:** de novo nucleotide synthesis, pyrimidine, intracellular alkalosis, pH deregulation

## Abstract

Replication is a fundamental aspect of cancer, and replication is about reproducing all the elements and structures that form a cell. Among them are DNA, RNA, enzymes, and coenzymes. All the DNA is doubled during each S (synthesis) cell cycle phase. This means that six billion nucleic acids must be synthesized in each cycle. Tumor growth, proliferation, and mutations all depend on this synthesis. Cancer cells require a constant supply of nucleotides and other macromolecules. For this reason, they must stimulate de novo nucleotide synthesis to support nucleic acid provision. When deregulated, de novo nucleic acid synthesis is controlled by oncogenes and tumor suppressor genes that enable increased synthesis and cell proliferation. Furthermore, cell duplication must be achieved swiftly (in a few hours) and in the midst of a nutrient-depleted and hypoxic environment. This also means that the enzymes participating in nucleic acid synthesis must work efficiently. pH is a critical factor in enzymatic efficiency and speed. This review will show that the enzymatic machinery working in nucleic acid synthesis requires a pH on the alkaline side in most cases. This coincides with many other pro-tumoral factors, such as the glycolytic phenotype, benefiting from an increased intracellular pH. An increased intracellular pH is a perfect milieu for high de novo nucleic acid production through optimal enzymatic performance.

## 1. Introduction

Folic acid was synthesized in 1937, and it was identified as a necessary factor for bone marrow function. Interestingly, folate-depleted feeding produced bone marrow damage similar to mustard gas. Sidney Farber, a pediatric pathologist working in Boston, used folic acid to treat anemia in children with acute leukemia. The results were devastating. Leukemia lit up and progressed very quickly. Farber concluded that it was necessary to find a substance that could do the opposite: deplete folate. He found the right chemist, Yellapragada Subbarao, who developed aminopterin, a folate antagonist that induced remissions in children with acute leukemia. Thus, in 1948–1949, the first anti-metabolite was found [1,2,3,4,5]. Interestingly, the first antimetabolite directly targeted nucleic acid synthesis. However, Farber and all the oncologists who adopted aminopterin as the gold standard for pediatric leukemia in the 1950s did not know how it worked beyond folate antagonism nor did they know anything about nucleic acid synthesis. It took the discovery of the structure of DNA in 1953 and painstaking biochemical research afterward to find out how nucleic acids are synthesized. This also led to the development of new inhibitors. This short saga about folic acid antagonists shows that a drug can be used successfully even if its mechanism of action is unknown. However, at the same time, ignoring the mechanism can cause unexpected damage. Understanding how nucleic acids are synthesized is necessary if we expect to inhibit their production.

A separate issue was the finding that the intracellular pH (pHi) in tumors is more alkaline than in normal counterparts [6]. In addition, it has been known since the 1970s that most animal eggs become more alkaline immediately before mitosis [7,8,9,10]. In this regard, it was found that cytoplasmic pH controls protein synthesis in fertilized eggs and early embryos [11]. In 2000, Reshkin et al. discovered that one of the first events in the cellular transformation was increased pHi [12].

This review will analyze the enzymes that participate in the synthesis of nucleic acids, particularly pyrimidines, and how intracellular pH interacts with them. Understanding this relationship may help develop new pharmaceuticals and improve the results of those already in use. This analysis will show that an increased intracellular pH seems to be a facilitator, if not a condition, for the synthesis of pyrimidines, thus generating both DNA and RNA.

### 1.1. Nitrogen Bases Nucleotide Synthesis

**Figure 1:**
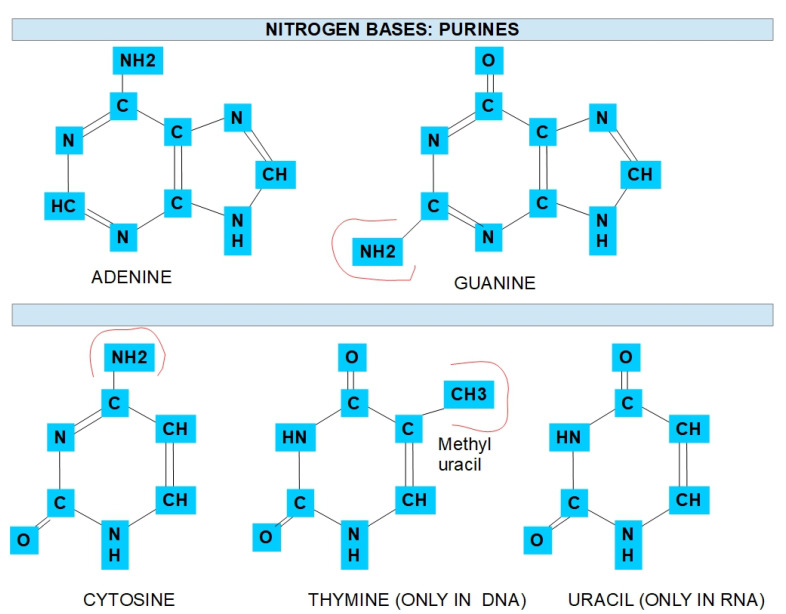
Chemical structure of nitrogen bases.

Cell division requires adequate nucleotide pools for increased DNA and RNA production in highly proliferative cells. The DNA building blocks consist of pyrimidines, purines, deoxyribose, and phosphate. Two types of nitrogen bases form part of DNA and RNA: purines and pyrimidines (Figure 1).

According to the Chargaff rule (1948), there are as many pyrimidines as purines in DNA [5], the first hint towards the base paring system (a purine always pairs with a pyrimidine and vice versa) that, a few years later, allowed Watson, Crick, Wilkinson, and Franklin to explain the DNA structure. However, in 1953, when this seminal discovery was published, nothing was known about how these nitrogen bases were synthesized. While existing pyrimidines and the re-utilization of pyrimidines from the degradation of some cell structures are sufficient for the resting cell, the situation is completely different in malignancies and highly proliferating cells, where new pyrimidines need to be developed [13].

There are two pathways for nucleotide synthesis [14]:

1. The de novo pathway starts with the precursor molecules, such as amino acids, CO_2_, NH_3_, and the sugar ribose-5-phosphate. Usually, proliferating and non-proliferating cells preferentially use the de novo pathway rather than the salvage mechanism [15,16].
2. The salvage pathway is a process of recycling existing nucleotides and bases that originated in the breakdown of existing nucleic acids.

In this review, we focus only on the de novo synthesis of the pyrimidine structure. The de novo synthesis of pyrimidines and purines is similar in all living beings.

A few basic but essential concepts:

▶ In purine and pyrimidine biosynthetic pathways, an amino acid is a precursor of each path:
  ■ Glycine for purines.
  ■ Aspartate for pyrimidines.
▶ Glutamine is the source of amino groups.
▶ In purine and pyrimidine de novo pathways, many enzymes are organized as large multi-enzyme complexes.
▶ In both purine and pyrimidine de novo synthesis, a negative feedback loop regulates the number of molecules to be synthesized.
▶ The cellular pools of nucleotides are generally very small compared to the amount needed for DNA or RNA synthesis. Thus, nucleotide synthesis is an essential process for cell replication and growth. This becomes even more evident in highly proliferating cells, such as those found in tumors.
▶ Limiting nucleotide synthesis decreases proliferation and growth.
▶ Drugs that can inhibit nucleotide synthesis can impede, delay, or decrease malignant proliferation.
▶ Pyrimidines are mainly produced by tumor cells, but stromal cells, such as macrophages and cancer-associated fibroblasts, can also produce them. In pancreatic cancer, it has been found that pyrimidines produced by macrophages were able to create resistance to gemcitabine treatment [17].
▶ Glutamine transporters that provide glutamine to the cell (glutamine is a nitrogen donor for pyrimidines) alkalinize the intracellular milieu by simultaneously exporting protons [18].

### 1.2. Pyrimidine De Novo Synthesis

**Figure 2:**
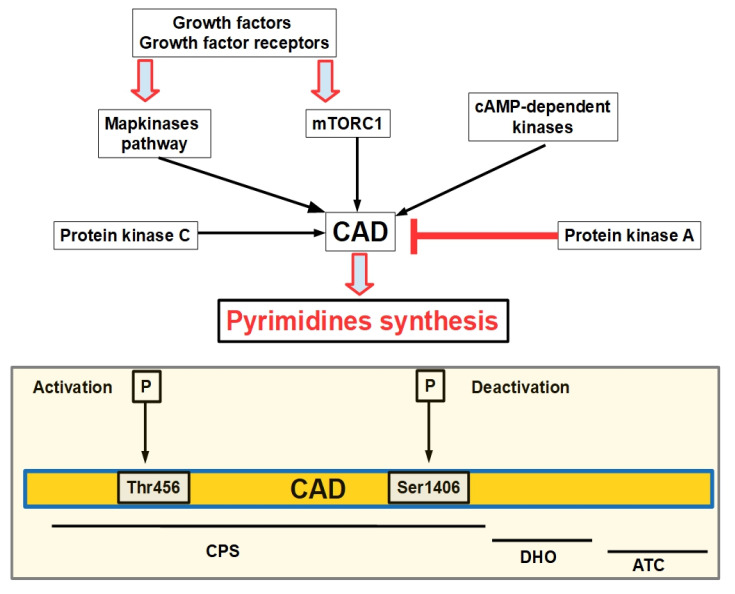
Signaling pathways that control CAD activity. This diagram is based on references [20,21,22,23]. CAD initiates de novo pyrimidine synthesis. CAD is activated by the activation of growth factors binding growth factor receptors and triggering the MAP kinases pathway. This activation takes place at the beginning of the S phase in the cell cycle. After the S phase is over, CAD is deactivated by protein kinase A (PKA) phosphorylation. The lower panel shows that phosphorylation of the Thr456 residue activates the enzyme, while phosphorylation of Ser1406 acts in the opposite way. The diagram also shows the three enzymes that form CAD. In this regard, the activation and deactivation residues form part of the first enzyme that initiates synthesis, namely, CPS (carbamoyl phosphate synthetase). DHO: dihydroorotase; ATC: aspartate transcarbamoylase.

Pyrimidine nucleotides are essential building blocks not only for nucleic acid synthesis but DNA repair and other cell functions as well [19]. Pyrimidine biosynthesis is initiated and regulated by a multienzymatic complex, CAD (CPS-II, aspartate transcarbamoylase, and dihydroorotase), that harbors the enzymes required for the first three steps of biosynthesis. This association of enzymes in one big polypeptide is an unusual finding in superior eukaryotes. This explains the difficulties found in characterizing and defining the properties of each of the enzymatic domains in a separate manner. In addition, CAD self-assembles in hexameres. CAD, which controls the pathway, is in turn, controlled by pro-proliferation and pro-biosynthesis pathways (Figure 2).

## 2. The Pyrimidine Synthesis Pathway

**Figure 3:**
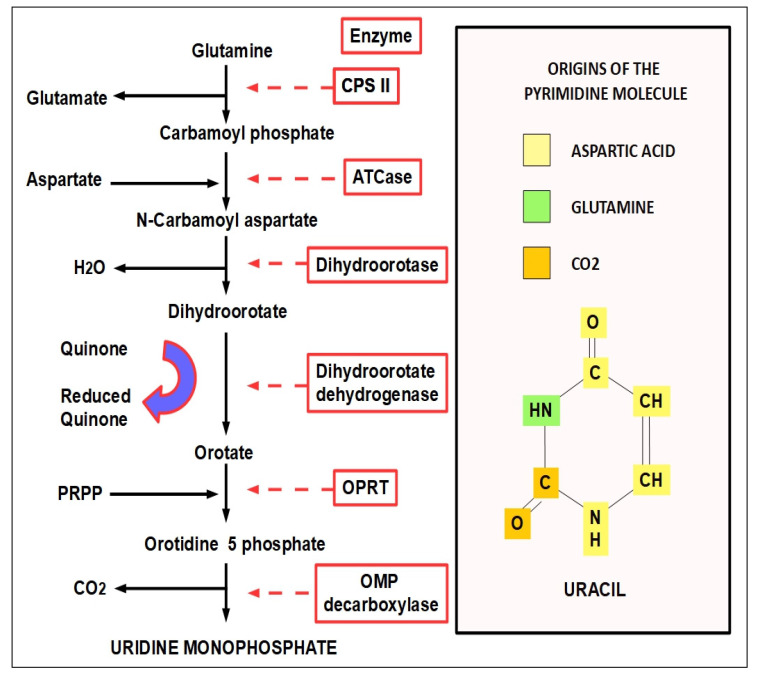

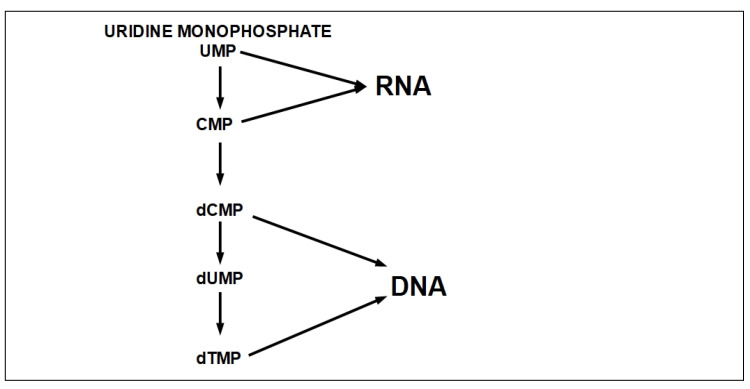
All the steps of de novo pyrimidines synthesis (**left** panel). The participating enzymes are in red frames. The **right** panel shows the origin of the different parts that form the pyrimidine structure. Uracil is being used as an example.

Figure 3 shows a full view of the de novo synthesis of pyrimidines from their origin in glutamine up to the pyrimidine uridine monophosphate. The diagram shows the origin of the different parts of a uracil molecule, which receives contributions from aspartic acid, glutamine, and CO_2_ (converted into HCO^3−^ by cytoplasmic carbonic anhydrases). The major amino acid contributors are aspartic acid and glutamine. Each of them also donates an amino group. Glutamine contributes to an NH_2_ group (see right panel of Figure 3).

CPS II: carbamoyl phosphate synthetase II; ATCase: aspartate transcarbamoylase; OPRT: orotate phosphoribosyltransferase; PRPP: phosphoribosyl diphosphate (PRPP). This whole synthetic process consumes seven ATP molecules.

### 2.1. The Steps in De Novo Pyrimidine Synthesis

**Step 1:**

**Figure 4:**
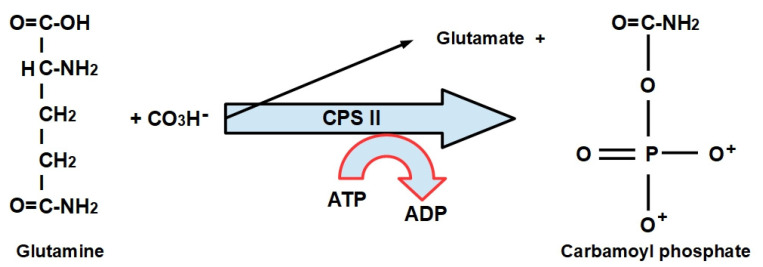
Reaction catalyzed by carbamoylphosphate synthase II.

In the presence of ATP, L-glutamine and bicarbonate are converted to carbamoyl phosphate (CAP) by carbamoyl phosphate synthetase II (CPS-II) in the cytoplasm (Figure 4).

There are two types of CPS:

(1) CPS-I is an intra-mitochondrial enzyme;
(2) CPS-II is cytosolic and is the enzyme that participates in de novo pyrimidine biosynthesis. This is the rate-limiting enzyme in pyrimidine biosynthesis.

CPS-II is upregulated in many cancers [24], and mainly in hepatoma [25,26,27]

The optimal pH for CPS-II activity is 7.4 [28]. Normal cells have an intracellular pH between 7.1 and 7.2. This means that to achieve an efficient function of this rate-limiting enzyme, the cytoplasm requires a higher pH. Interestingly, tumors raise their intracellular pH to 7.4–7.5 [29]. The difference seems small, but it must be remembered that pH is a logarithmic function, thus the difference represents an important increase in proton concentration. When the pH reaches the optimal point for an enzyme, this means that the enzyme is working at its maximum efficiency and speed.

The end product of the events leading to pyrimidine synthesis is uridine monophosphate (UMP), which inhibits CPS-II via a negative feedback loop (not shown in Figure 3).

**CPS-II activators.** Many activators, such as ATP and phosphoribosyl diphosphate (PRPP), stimulate the expression of this enzyme [30]. mTORC1 is a key modulator that upregulates the whole pathway [31] and, through its downstream protein S6K1, induces the expression of all the enzymes involved in de novo pyrimidine synthesis, including CPS-II [32] and the other two enzymes that participate in steps 2 and 3. Actually, the three enzymes that catalyze the first three steps of pyrimidine synthesis, namely, CPS-II, aspartate transcarbamoylase, and dihydroorotase, form an enzymatic complex that is known by the acronym CAD, as mentioned above.

**CPS-II Inhibitors.** CPSII is inhibited by acivicin, an analog of glutamine [33]. In this respect, a glutamine-restricted diet might limit pyrimidine biosynthesis, interrupting the nucleic acid synthesis.

**Step 2:**

**Figure 5:**
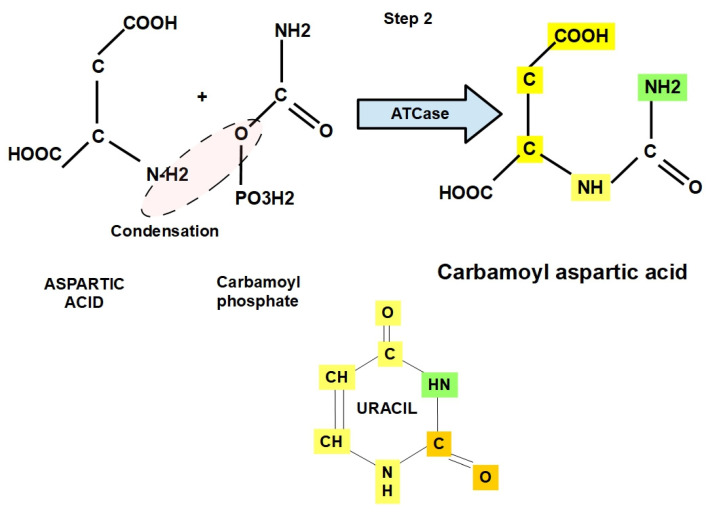
Condensation between aspartic acid and carbamoylphosphate, generating carbamoyl aspartate through the enzymatic action of ATCase (aspartate transcarbamoylase). The structure of uracil is shown in the lower panel for a comparative view of how this structure is being built.

The carbamoyl phosphate (CAP) interacts with aspartic acid, generating carbamoyl aspartic acid (CAA) through condensation via aspartate transcarbamoylase (ATCase) (Figure 5).

The impact of pH on ATCase (aspartate transcarbamoylase) activity depends on the substrate concentration: the optimal action occurs at an alkaline pH of 8.5 in bacteria [34] and at around 9 in rat liver [35]. Here again, we find that a high intracellular pH favors the synthesizing steps.

There is strong evidence showing that ATCase is upregulated in tumors, and its down/regulation by *N*-(phosphonacetyl)-l-aspartate (PALA) inhibits tumor growth [36,37,38,39,40]. PALA may have antitumor activity and potentiates the effects of chemotherapeutic agents, e.g., 5-fluorouracil (5-FU) [41]. Moreover, 2-phenyl-1,3-4(H)benzothiazin-4-thione (quinazolinone derivative) is a strong ATCase inhibitor [42]. The pyrimidine pathway might form cytidine triphosphate (CTP); this CTP blocks ATCase, reflecting its negative feedback mechanism. ATP activates ATCase.

**Step 3:**

**Figure 6:**
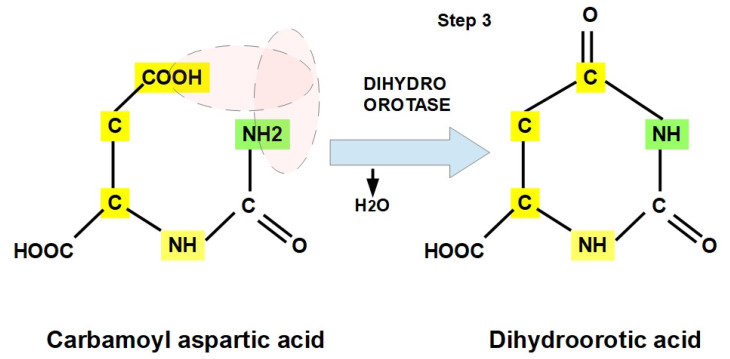
Third step. Conversion of carbamoylaspartate into dihydroorotate through the enzymatic action of dihydroorotase.

Carbamoyl aspartic acid (CAA) is converted to dihydroorotic acid (DHOA) via dihydroorotase (EC 3.5.2.3), which is also known as dihydroorotate hydrolase, and carbamoylaspartic dehydrase. Dihydroorotase is a metalloenzyme that catalyzes the reversible conversion of carbamoyl aspartic acid into dihydroorotic acid and closes the ring (Figure 6).

The effect of pH on dihydroorotase in cancer cell lines is a controversial and debated issue. However, the current concept is that dihydroorotase is driven forward by an acidic pH, while the reversible reaction (e.g., the formation of CAA) reaches its optimal efficiency at an alkaline pH [43,44,45,46,47,48].

At this point, the three enzymes forming CAD have catalyzed the ring structure that will form pyrimidines in the next steps from glutamine, CO_2_, and aspartate. The optimal pH for the first two enzymes is above 7.4, while the third enzyme requires a lower pH (around 7 or lower).

Hypoxia-inducible factor 1 alpha (HIF-1 alpha) and deferoxamine, metal chelator inducing HIF-1α expression, show inhibitory effects on the CAD multienzymatic complex [49]. This seems paradoxical because HIF-1 alpha is a strong tumor driver.

Therefore, although the dihydroorotase appears to be an enzyme that does not enhance the pyrimidine biosynthetic pathway, some data suggest that the same CAD also supports tumorigenesis [50]. However, it was also found that CAD downregulates the Wnt/Beta catenin pathway in colon cancer cells, decreasing migration [51].

### 2.2. Dihydroorotase Inhibitors

Dihydroorotase seems to interact with the thymidylate synthase inhibitor 5-fluorouracil [52]. However, this needs further research. Plumbagin, a natural product obtained from the carnivorous plant *Nepenthes miranda,* has shown strong competitive inhibition, causing apoptosis in tumors [52]. Analogs of carbamoyl aspartate have also shown inhibitory effects [53].

**Step 4:**

Dihydroorotic acid (DHOA) is converted to orotic acid (OA) via dihydroorotate dehydrogenase. This is the only redox reaction in the de novo biosynthesis pathway of pyrimidine nucleotides.

**Figure 7:**
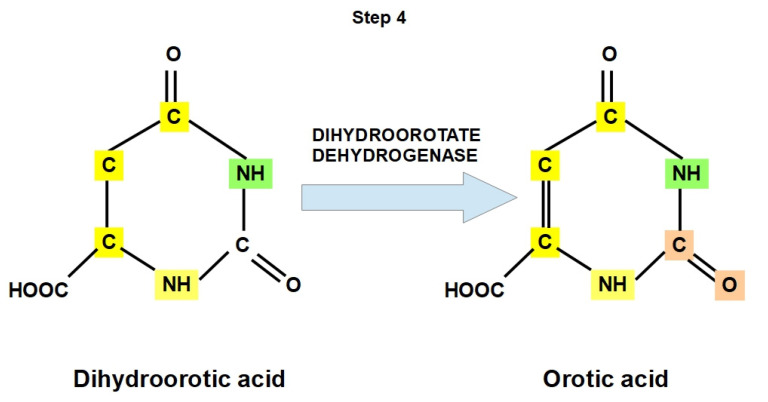
Step 4. Chemical reaction catalyzed by dihydroorotate dehydrogenase. The pyrimidine ring is initially formed as orotate. Then, in the next step (step 5), it is attached to ribose phosphate (which is generated in the pentose phosphate pathway) and finally converted to the pyrimidine nucleotides that will be used for DNA and RNA synthesis.

There are two dihydroorotate dehydrogenases, namely, class 1 (cytosolic) and class 2 (found in the inner mitochondrial membrane). In different organisms, dihydroorotate dehydrogenases use either NAD^+^, fumarate, or NADP^+^ as electron acceptors, yielding NADH, succinate, or NADPH, respectively [54,55,56,57,58]. Class 2 uses quinone as an electron acceptor, yielding hydroquinone [59,60,61,62,63] (Figure 7).

The optimal pH for this is around 8 [64,65]. This is logical because this enzyme carries out its work in the mitochondria [66], which have a higher pH than the cytoplasm.

Leflunomide is a drug used to treat rheumatoid arthritis that has shown significant efficacy against dihydroorotate dehydrogenase [67], and it could be considered as an anticancer drug candidate [68,69,70].

Other inhibitors, such as brequinar sodium, atovaquone, and ML390. have also been identified (See Box 1).

Box 1 **Brequinar.**

Brequinar sodium (a quinoline-carboxylic acid sodium salt) is a small molecule that acts as a specific inhibitor of dihydroorotate dehydrogenase, the fourth enzyme of the de novo pyrimidine biosynthetic pathway. Brequinar sodium inhibits dihydroorotate dehydrogenase that has ubiquinone as a cofactor but not when NAD is the cofactor [71].

Brequinar has shown a dose-dependent antineoplastic and immunosuppressant activity against different tumor models [72] and went through phase I and II clinical trials during the 1990s [73,74,75,76]. A narrow therapeutic window and high myelotoxicity led this drug to oblivion. In 2018, new studies confirmed that brequinar was not active at lower doses in solid tumors but was quite effective in leukemia [77]. This different behavior in non-solid tumors seems to confirm two concepts:

▶ Solid tumors have a higher level of pyrimidines that require toxic doses of brequinar, while leukemias respond to lower doses due to a decreased pool of uridine;
▶ The pyrimidine synthetic pathway is somehow related to the inhibition of myeloid differentiation because the inhibition of dihydroorotate dehydrogenase overcame a differentiation blockade in acute myeloid leukemia in vivo [78].

Interest in brequinar as a complementary drug for cancer treatment in both non-solid and solid tumors has been revived after 20 years [79,80,81,82,83]. The fact that brequinar has shown the ability to induce ferroptosis and to increase the ferroptotic effect of drugs such as sulfasalazine [84] is particularly interesting.

**Step 5:**

Orotic acid (OA) interacts with phosphoribosyl pyrophosphate to produce orotidine 5′-monophosphate (OMP), also known as orotidylic acid. The enzyme that catalyzes the reaction is called orotate phosphoribosyltransferase (OPRT).

**Figure 8:**
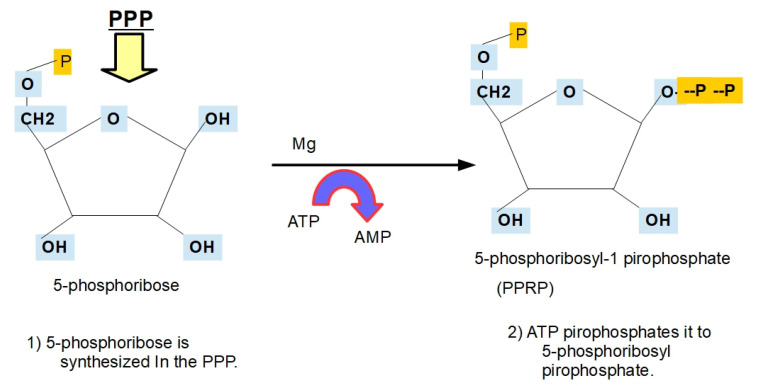
The origin of the sugar molecule of ribonucleotides.

**Figure 9:**
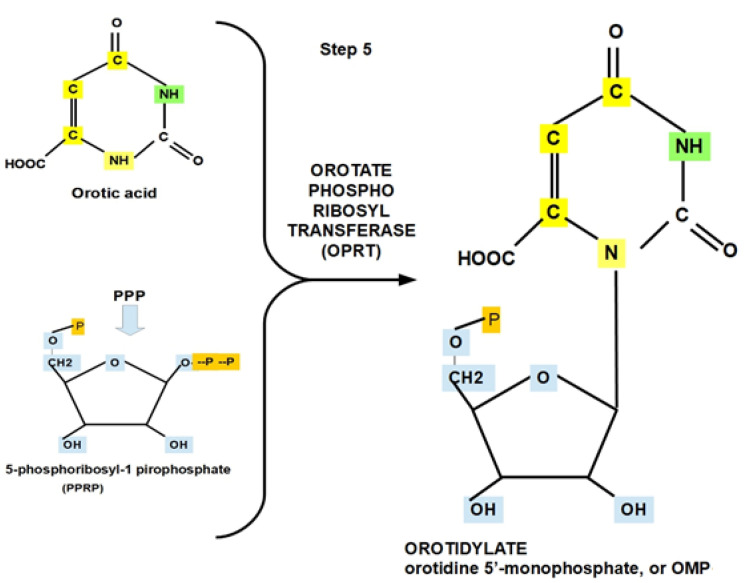
Binding of orotic acid with PPRP generating OMP through the enzymatic activity of OPRT.

Phosphoribosyl pyrophosphate originates in the first part of the oxidative phase of the pentose phosphate pathway [85]. Therefore, this step of the pentose phosphate pathway (PPP) branches to merge and/or amalgamate with de novo pyrimidine biosynthesis. Interestingly, there is a shared regulation of the glycolytic and PPP pathways by intracellular pH pHi. Indeed, as with glycolysis, the optimal activity of the enzymes driving the PPP occurs at an alkaline pHi, which is compatible with the cytoplasmic pH of cancer cells (Figure 8 and Figure 9).

Importantly, OPRT is an enzyme that contributes to the conversion of 5-FU into fdUMP, which is the active form of 5-FU [86]. Resistance to 5-FU seems to be associated with low OPRT expression [87,88]. OPRT enhances the chemotherapeutic response to 5-fluorouracil (5-FU) [89,90].

OPRT is overexpressed in many tumors, such as bladder cancer [91], and its expression is correlated with recurrence. Further, OPRT is useful as a marker of prognosis and for predicting chemosensitivity [90,92,93].

The optimal pH of the OPRT for the forward reaction is 8, while for the reverse reaction it ranges between 6.5 and 7.5 [94].

OPRT activity inhibitors include xanthosine, uridine 5′-phosphates, cytidine barbiturate, 5-flouro orotate (most effective), and higher concentrations of nucleotides [95,96]. OPRT inhibition also plays a role in other diseases, e.g., tuberculosis, toxoplasmosis, and malaria [97].

**Step 6:**

**Figure 10:**
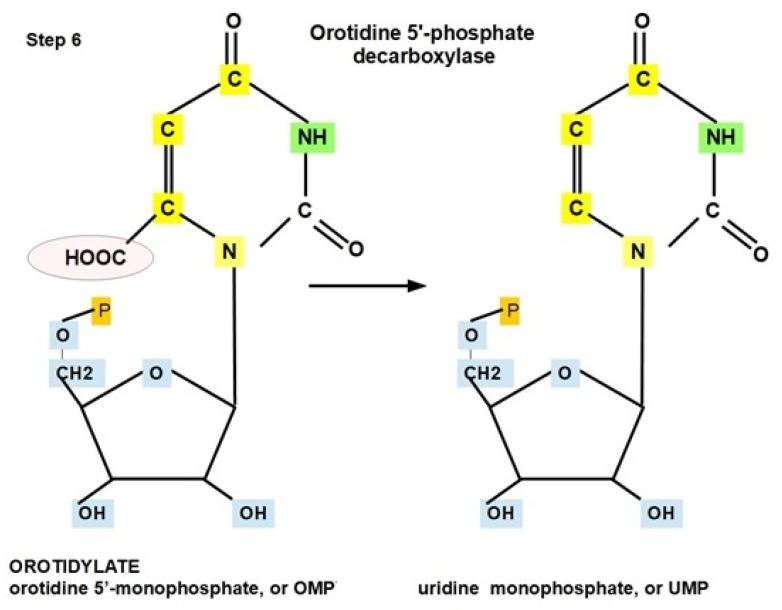
Decarboxylation of orotidylate to form UMP (uridine monophosphate, step 6).

Orotidine 5′-monophosphate is converted to uridine monophosphate (UMP) by orotidine 5′-phosphate decarboxylase (OMP decarboxylase, ODCase). As a result, OPRT plus OMP decarboxylase form the bifunctional enzyme called uridine monophosphate synthetase (UMPS) (Figure 10) [98].

There is not much information about the expression of ODCase in tumors. However, pyrazofurin and 6-Aza uridine 5′-monophosphate are examples of ODCase inhibitors with antitumor properties [99,100].

The optimal pH is approximately 7.5 [101,102].

**Step 7 (Uracil Formation):**

**Figure 11:**
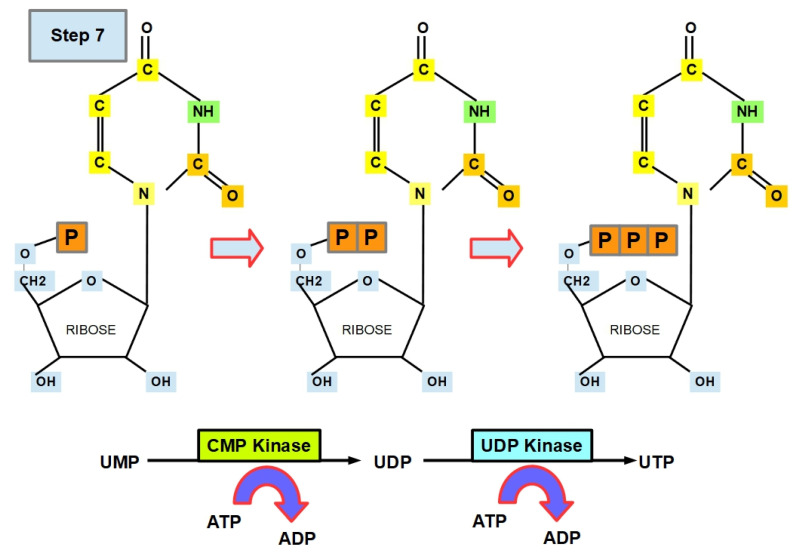
Phosphorylation of UMP generating UDP and phosphorylation of UDP generating UTP.

Two consecutive phosphorylations take place: steps 7.1 and 7.2 (Figure 11).

Step 7.1:

UMP is converted to uridine diphosphate (UDP) via cytidine monophosphate kinase (CMP kinase or uridine monophosphate-cytidine monophosphate phosphotransferase). CMP kinase is found in the cytoplasm, nucleus, and mitochondria [103,104,105].

The optimal pH for CMP kinase is 7.4 [106].

When CMP kinase 1 is overexpressed in cancer [107,108,109], it is a powerful indicator of poor prognosis. Importantly, the anticancer drug gemcitabine [108] inhibits CMP kinase [65].

Step 7.1 is a crossroad of de novo pyrimidine biosynthesis, and it can follow two main branches.

Step 7.2:

UDP is further phosphorylated to UTP using nucleoside-diphosphate kinase (UDP kinase). UTP can enter additional metabolic pathways, including glycogen, galactose, and glucosamine [110,111]. Moreover, it acts as a substrate (ligand) for purinergic receptors (P2 receptors) [112].

Here, the optimal pH is 8 [113].

NM23 (nucleoside-diphosphate kinase), which is known as a metastasis suppressor [114], produces nucleoside-diphosphate kinase, although in some tumors (e.g., melanoma, breast, and colon), its expression is associated with low metastatic potential. In others (neuroblastoma and osteosarcoma), it is related to a decrease in survival rate [115]. Therefore, its role is unclear. One of the possible explanations is that, while NM23 protein suppresses metastasis, it does not alter the growth of primary tumors [116].

Theophylline (phosphodiesterase inhibitor) inhibits NDK [117].

**Step 8 (Cytosine Formation):**

**Figure 12:**
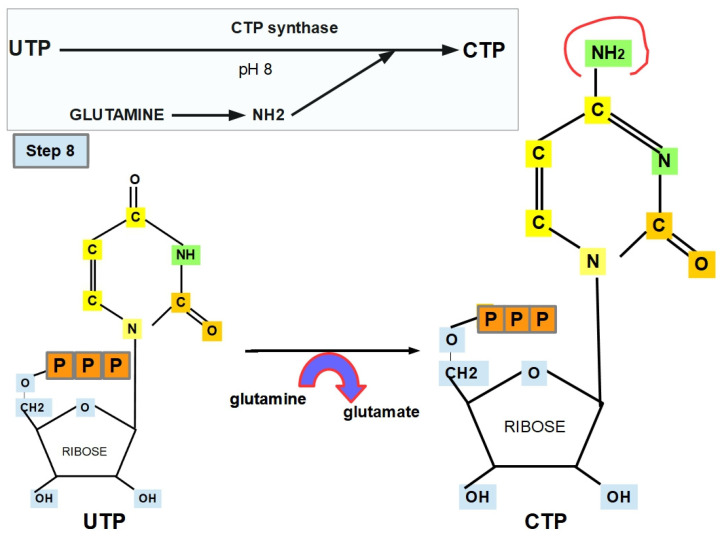
CTP synthesis.

UTP is converted to cytosine triphosphate via cytosine triphosphate synthase (CTP synthase), also known as UTP: ammonia ligase. Glutamine is also crucial in carrying out this step [118,119]. Therefore, the presence of glutamine is essential for starting de novo pyrimidine biosynthesis and forming cytosine (Figure 12).

The optimal pH of CTP synthase is around 8 [120].

CTP synthase expression is markedly increased in very rapidly growing tumors [121,122]. Moreover, a CTP synthase abnormality may be associated with resistance [123].

Cyclopentenyl cytosine is an inhibitor of CTP synthase [121], while GTP is a CTP synthase activator [120,122,124].

The steps discussed above show the synthesis of the two pyrimidine bases that form the nucleotides for RNA, namely, the uracil and cytosine nitrogen bases and the uridine and cytidine nucleotides, respectively.

**Step 9 (Thymine Formation):**

**Figure 13:**
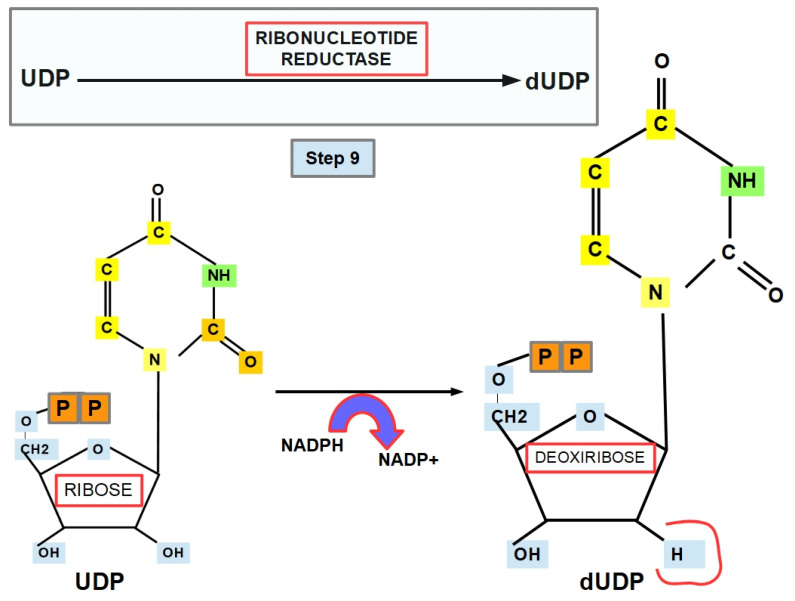
Step 9: The sugar, ribose, is reduced to deoxyribose, forming dUDP.

DNA has thymine instead of uracil. In this step, UDP is reduced to produce dUDP (deoxyuridine diphosphate) via ribonucleotide reductase, which converts NADPH to NADP^+^ (Figure 13).

**Step 10:**

**Figure 14:**
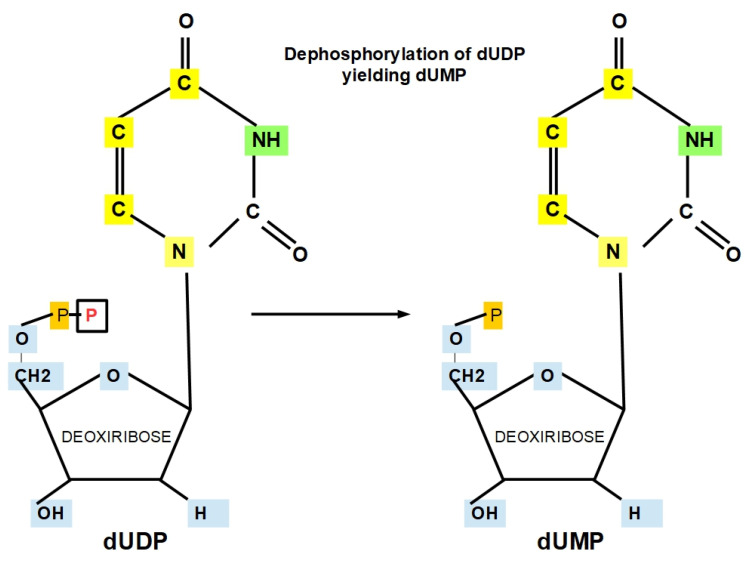
Dephosphorylation of dUTP yielding dUMP.

dUDP is dephosphorylated, yielding dUMP (deoxyuridylic acid or deoxyuridylic acid or deoxyuridylate) (see Figure 14).

Steps 9 and 10 depend on NADPH and its possible cellular level; therefore, this reaction might occur in the cytoplasm rather than the nucleus. Furthermore, it might interact with additional pathways, e.g., the pentose phosphate pathway (PPP) and the methylglyoxal pathway [85,125]. Therefore, it raises more critical questions: Does de novo pyrimidine biosynthesis occur in the nucleus or cytoplasm or even in the mitochondrion? Does the nucleus have its own de novo pyrimidine biosynthesis pathway, while the mitochondrion has a separate biosynthetic pathway?

Because this step supports DNA synthesis, NADPH is crucial for DNA synthesis.

Ribonucleotide reductase (RR), also known as ribonucleoside diphosphate reductase (rNDP), is a ubiquitous cytosolic enzyme with an optimal pH of around 7.5 [126].

RR is expressed in many tumors, e.g., breast, pancreas, lung, adrenal cortex, etc., and it is a prognostic marker and a therapeutic target [127,128,129,130,131,132,133,134].

Some examples of RR inhibitors are (-)-epicatechin 2-aminobenzohydroxamic acid, 5′-*O*-valproyl-3′-C-methyladenosine, cisplatin, chlorambucil, desferrioxamine, gemcitabine, and hydroxyurea [135,136,137,138,139,140,141]. It has also been shown that H_2_O_2_ and glutathione have an inhibitory effect on RR in mice [139,142]. On the other hand, ATP is an RR activator [140,143,144], while p53 also activates RR during the DNA repair process [145].

**Step 11:**

**Figure 15:**
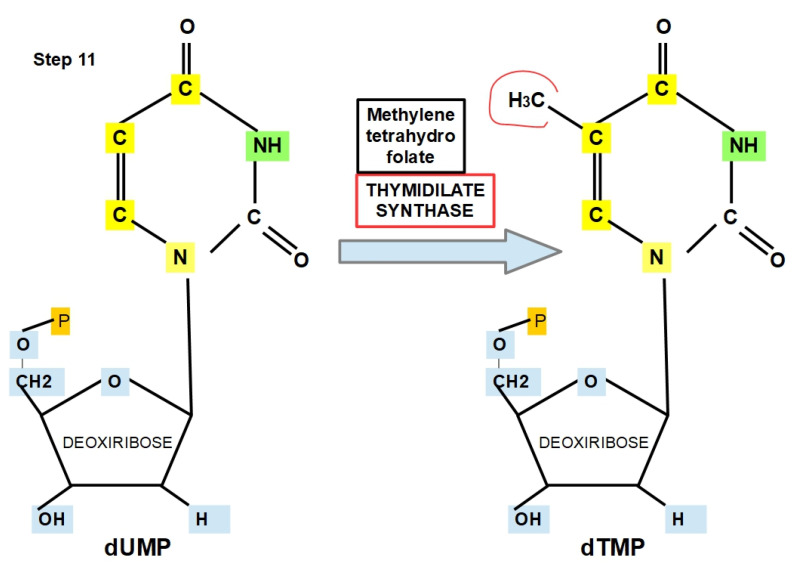
Thymidilate synthase as the enzyme and methylentetrahydrofolate as cofactor add a methyl group on the nitrogen base. This converts dUMP to dTMP. Two further phosphorylations generate dTTP.

dUMP is converted to dTMP via thymidylate synthase (TS), which requires 5,10-methylenetetrahydrofolate as a co-enzyme. Then, dTMP can be phosphorylated to dTDP and dTTP, which is the molecule used for DNA synthesis (Figure 15) [146].

The optimal pH range in leukemia is 7.0–8.1 [147].

TS is also a prognostic biomarker in tumors and is associated with drug resistance [148,149,150,151,152,153].

Capecitabine and 5 fluorouracil are examples of TS inhibitors [154,155,156,157].

## 3. Discussion

The de novo biosynthesis of pyrimidine is a complex process. Each step of the biosynthetic pathway is influenced by many factors. Part of these factors includes the redox state of the cell NADPH/NADP^+^, ATP, GTP levels, the optimal pH for the related enzymes, the availability of glutamine and aspartate among others. The synthesis of cytosine and uracil might occur at a higher pH compared to thiamine.

Pyrimidine is not limited to the biosynthesis of nucleic acid. It also plays crucial roles in tumor metabolism. Therefore, can pyrimidine act as an oncometabolite?

Interestingly, the optimal pH of most enzymes participating in this pathway is slightly higher than that of normal cells. An increased intracellular pH is usual in malignancies. Table 1 shows these optimal pH levels and in all cases they are above 7.3, except for thymidylate synthase. The protumoral effects of an alkaline or hyperalkaline intracellular milieu has been extensively investigated and confirmed [6,158,159].

For example, in the case of ribonucleotide reductase (RR), the key enzyme to produce nucleotides for DNA, indirect evidence shows that the optimal pH is between 7.5 and 8. These data come from the fact that at this pH range less iron is needed for the maximum activity of the enzyme [160]. However, with high levels of iron, the optimum pH is in the range of 6.5–7 [161]. RR requires iron and folic acid for its adequate functioning. This case shows the difficulties in correctly determining the optimal pH of many of the enzymes involved in nucleotide synthesis. Furthermore, tumors are high iron consumers.

Hormesis is a biphasic pharmacological dose-dependent response characterized by stimulation by a low dose and inhibition by a higher dose [162,163]. Moreover, hormesis is a redox-dependent process that might support cell death (apoptosis), such as with reactive oxygen species (ROS), an example of a stressful condition; however, the release of ROS at different concentrations supports genetic mutations.

Heat shock proteins (HSPs) are proteins (molecular chaperones) synthesized in response to stressful conditions. They play a crucial role in protein maturation and folding, so they are defense proteins that support cellular survival in stressful conditions. Therefore, it will not be surprising if many tumor cells are found to be HSP-dependent. It has been shown there is an association between HSPs, cancer, and pyrimidine synthesis. Hence, one of the future directions in managing cancer is further studying the de novo pyrimidine biosynthesis–HSP interactions and the potential opportunity that offers a new therapeutic window in managing cancer [164,165].

This review shows another face of pH deregulation and its link with increased proliferation. Hypothetically, we may assume that by lowering intracellular pH the activity of these enzymes may be slowed down, thus decreasing pyrimidine synthesis. Table 1 also shows the possible inhibitors for each enzyme.

## 4. Conclusions

1. Cancer cells have an increased rate of pyrimidine synthesis, as expected in a cell that must duplicate all its DNA and RNA at an accelerated pace.
2. The enzymes participating in pyrimidine synthesis have an optimal efficiency at a pH that is higher than the intracellular pH of normal cells.
3. This increased intracellular pH is constantly found in malignant cells as part of the pH paradigm.
4. On a theoretical basis, we may assume that lowering the intracellular pH will hamper the efficiency of pyrimidine synthesis and decrease tumor proliferation. There is direct and indirect evidence that intracellular acidification is a valid method for complementing standard treatment schemes.

## Figures and Tables

**Table 1:** The key enzymes that participate in the de novo biosynthesis of pyrimidine.

Enzyme	Optimal pH	Possible Inhibitor
Phosphate synthetase II	7.4	Acivicin [33]
Aspartate transcarbamoylase (ATCase)	pH in bacteria (8.5)	*N*-(phosphonacetyl)-l-aspartate (PALA) [38]
Dihydroorotate dehydrogenase	8	Leflunomide [68,69,70]
Orotate phosphoribosyltransferase	The forward reaction is 8, the reverse one is 6.5–7.5	Include xanthosine, uridine 5′-phosphates, cytidine barbiturate, 5-flouro orotate [95,96]
Orotidine 5′-phosphate decarboxylase	approx. 7.5	Pyrazofurin and 6-aza uridine 5′-monophosphate [99,100]
Cytidine monophosphate kinase	7.4	Gemcitabine [65]
Nucleoside-diphosphate kinase	8	Theophylline [117]
Cytosine triphosphate synthase	8	Cyclopentenyl cytosine [121]
Ribonucleotide reductase enzyme	7.5–8	Cisplatin, chlorambucil, desferrioxamine, gemcitabine, and hydroxyurea [135,136,137,138,139,140,141]
Tymidylate synthase	7.0 and 8.1	Capecitabine and 5 fluorouracil [154,155,156,157]
Ribonucleotide reductase	7.5 to 8 with a low iron level	Gemcitabine and iron chelators [160]

## Data Availability

Not applicable.
